# Fundamental data regarding the emergency management of infectious diseases in higher education institutions: HIV knowledge and stigma among college students in Southwest China

**DOI:** 10.3389/fpubh.2026.1970246

**Published:** 2026-10-15

**Authors:** Fang Pei, Xin Li, Hao Yao, Rong Pei

**Affiliations:** 1 School of Economics and Management, Civil Aviation Flight University of China, Guanghan, Sichuan, China; 2 Sichuan Provincial Engineering Research Center of Smart Operation and Maintenance of Civil Aviation Airports, Guanghan, Sichuan, China; 3 School of Public Health, Chengdu University of Traditional Chinese Medicine, Chengdu, China

**Keywords:** college students, emergency management, HIV, HIV knowledge, sexual violence, stigma

## Abstract

This study aimed to assess the knowledge on human immunodeficiency virus (HIV) and factors related to HIV stigma among college students to enlighten them on the development of HIV prevention strategies and better understand the additional benefit of HIV risk reduction, supplied fundamental data for the emergency management of infectious diseases in higher educational institutions. This cross-sectional study enrolled students from 10 universities in southwest China (*n* = 4,133). Survey questions included demographic information, HIV knowledge, HIV stigma, and self-esteem. Descriptive and inferential statistics were applied to summarize the data. Logistic regression was used to analyze factors related to HIV knowledge and stigma. The knowledge level of respondents on HIV was 74.2%. Stigma was highly prevalent in the form of fear of casual transmission (79.8%), moral judgment (71.1%), personal stigma (72.2%), and perceived community stigma (71.8%). The findings demonstrated that a high level of relevant knowledge regarding HIV was consistently associated with lower stigma levels in four dimensions, and that seven variables—, namely, gender, ethnicity, grade, only-child or not, sexual orientation, awareness of the HIV/acquired immunodeficiency syndrome epidemic, and self-esteem—, played different roles in affecting various dimensions of HIV stigma. The level of HIV knowledge was low whereas HIV-associated stigma was high among college students in southwest China. Addressing HIV in colleges should incorporate efforts for reducing stigma associated with HIV. For effective infectious disease emergency management in academic settings, this study underscores the necessity of integrating stigma-reduction strategies into institutional preparedness plans. Addressing these barriers is essential for optimizing risk communication, intervention uptake, and response efficacy during public health emergencies.

## Introduction

Despite the tremendous biomedical advances in the recent years that have made HIV more treatable, human immunodeficiency virus (HIV) infection continues to be a major threat to public health worldwide (1). College students are in an age group having the highest probability of acquiring HIV (2). According to the Chinese Center for Disease Control and Prevention (CDC), HIV infection among college students is increasing, with an annual growth rate of the number of newly diagnosed college students ranging 30%−50% over the past several years (3). This escalating risk within the dense, dynamic environment of higher education institutions (HEIs) underscores the critical need for robust emergency management of infectious diseases in these settings.

HIV testing services are a part of the major HIV responses to prevent onward sexual transmission of HIV and reduce risky behaviors (4). In China, free HIV testing is available as part of an HIV prevention campaign charge (5). However, the majority of college students have never been tested for HIV despite having unprotected sex and multiple sexual partners (6–8). This information indicated that HIV prevention strategies must be enhanced to minimize the spread of infection, especially among those who are unaware of their HIV status (9). Strengthening these strategies is essential for mitigating outbreaks and enhancing institutional preparedness.

Stigma associated with HIV is a substantial hindrance to combating the HIV and acquired immunodeficiency syndrome (AIDS) epidemic (10). Individuals may develop stigma owing to misleading information about HIV transmission and judgmental outlooks toward individuals who are disproportionately affected by HIV, such as men who have sex with men, ethnic minorities, and injection drug users (11). In addition, the gaps in HIV knowledge as well as lack of access to information may cause stigma to people living with HIV/AIDS (PLWHA) (9). Many studies have shown that HIV-related stigma was associated with decreased HIV testing and reduced efficacy of HIV-preventive behaviors, such as condom usage (12, 13).

Discrimination and stigma negatively affect people with HIV infection, which is a broad and global social phenomenon (11). Several studies have reported the association between HIV-related and sexual stigmas (14, 15). Therefore, HIV-related stigma may affect the sexual attitudes of college students. One of the major factors that contribute to stigma is poor knowledge regarding HIV, particularly knowledge on transmission (10). The stigma for HIV-positive individuals, may increase due to insufficient knowledge about HIV, unknown HIV status, and misconceptions about HIV transmission among college students (9). New strategies must be developed to prevent HIV infection among college students. With the aim to end the AIDS epidemic by 2030, planned global efforts must be undertaken on a large scale to reduce stigma (16).

In China, the impact of HIV-related stigma on its prevention and cure is prominently visible (13). Therefore, the purpose of this study is to provide fundamental data crucial for the emergency management of infectious diseases in higher education institutions. The purpose of this study was to measure the level of knowledge on HIV and stigma of college students with HIV in southwest China and evaluate the factors associated with stigma attitudes toward PLWHA. The study findings are expected to indicate ways to reduce stigma attitudes toward PLWHA among college students and to illuminate the gaps in HIV prevention strategies in the higher education system.

## Methods

### Study design

This study was an analytical, cross-sectional study conducted between October and December 2020 comprising 10 colleges in southwest China. All universities included in this study were located in Sichuan Province, which has the highest numbers of HIV-infected individuals in China (17). This study was approved by the Institutional Review Committee of the institution where the data were collected. All methods used in this study were conducted in accordance with relevant guidelines and regulations. The schools were selected through judgment sampling, and sample classes were then selected via stratified and cluster-sampling. All the selected students were required to answer a standardized self-administered questionnaire on the day of the survey. The questionnaires were distributed by the teachers at each school. Data on demographics, HIV knowledge, experiences of violence, sexual concepts, HIV stigma, risk behaviors, and HIV testing were collected. Overall, 4,133 students completed the survey with a response rate of 99%. The study aimed to generate fundamental data critical for informing emergency management protocols for infectious diseases within HEIs.

### Patient and public involvement

Before the survey questionnaire was sent out, participants were informed about the voluntary nature of participation, and that they could withdraw from the study at any time and remain anonymous. Ethics approval was obtained from the ethnic committee of Sichuan University of Arts and Sciences.

## Measures

### Sociodemographic variables

Participants were required to provide their age and gender, ethnicity (Han/others), family background, sexual orientation, sexual behavior, history of being tested for HIV testing, and whether they were aware of the HIV/AIDS epidemic in their region.

### HIV knowledge

Knowledge regarding HIV/AIDS was measured through eight items in the questionnaire which were specially developed by the National Center for AIDS/STD Control and Prevention, China CDC for college students (18). This scale evaluates knowledge on HIV transmission and prevention. For each item, the correct answer (“yes”) and wrong answers (“no” or “unknown”) were scored as 1 and 0, respectively. All correct responses were summed, those who had more than six correct answers on HIV/AIDS knowledge were considered to have a high HIV/AIDS knowledge level (18–20).

### HIV stigma

The main outcome of interest was HIV stigma. The Chinese version of Zelaya's HIV-related stigma scale was applied to evaluate HIV stigma in this study. This scale contains the following four categories with six items in each category: (1) fear of casual transmission, (2) moral judgment, (3) personal stigma, and (4) perceived community stigma. Participants were asked to indicate their extent of expressing fears or agreement when faced with these 24 situations, by selecting one of the three responses (Yes, No, Not sure). For all t items, “Not sure” responses were marked as incorrect (0), whereas “Yes” and “No” responses were marked correct (1). To achieve higher scores and reflect less biased attitudes, all items were reversed when it was appropriate. Each dimension was individually coded if the participant correctly responded to all items in their respective dimensions, which was 0 (lower stigma); otherwise, the score was 1 (higher stigma) after the final logistic regression analysis (21).

### Self-esteem

The Rosenberg self-esteem scale (SES) was used to measure the self-esteem of college students (22). The Chinese version of the SES has been widely used, including for teenagers, in the Chinese population (23). SES includes 10 questions (total score range 10–40). A higher score indicated higher self-esteem. An SES score of >27 points was considered to indicate high self-esteem.

### Statistical analysis

All analyses were conducted using Statistical Analysis Software SPSS 21.0 (2012; SPSS Inc., Chicago, IL, USA). No missing values were noted. Continuous variables were described in terms of the mean and standard error of the mean. Categorical variables were described in terms of the proportion and compared using the Pearson's χ^2^ or Fisher's exact tests. Moreover, stepwise selection method-using multivariate logistic regression models were used to determine the statistically significant variables affecting the different dimensions of HIV knowledge and stigma. Finally, we also calculated the adjusted odds ratios (AOR) and 95% confidence intervals (CI). A *p* value of < 0.05 was considered statistically significant.

## Results

### Characteristics of participants

A total of 4,133 participants were recruited in this study. The, effective data utilization rate was 99%. Participants were aged 16–28 years with a mean (standard deviation) of 19.45 (1.43) years. Moreover, 62% of the participants were female, and 47% were freshmen. Over 89% of the participants were Han Chinese, the predominant ethnic population in China. A total of 53.8% of the participants were from rural areas, and 29.2% were from one-child families. Similar to our previous study, 11.8% identified themselves as non-heterosexuals, and 9.1% admitted to engaging in sexual behaviors. Most participants (92.6%) had never been tested for HIV, and only a few were aware of the HIV/AIDS epidemic in their region (15.2%; Table 1).

**Table 1:** Socio-demographic and behavioral characteristics.

Characteristic	*N*	Percent
Gender		
Male	1,584	38.3%
Female	2,549	61.7%
Ethnicity		
Han	3,517	89.3%
Others	422	10.7%
Grade		
Freshmen	1,933	46.8%
Non-freshmen	2,200	53.2%
Home address		
Rural	2,225	53.8%
Town	1,908	46.2%
Only-child or not		
Yes	1,208	29.2%
No	2,925	70.8%
Sexual orientation		
Heterosexuals	3,646	88.2%
Non-heterosexuals	487	11.8%
Sexual behavior		
Yes	377	9.1%
No	3,756	90.9%
Whether had ever had HIV testing		
Yes	304	7.4%
No	3,829	92.6%
Whether aware of the HIV/AIDS epidemic in their region		
Yes	629	15.2%
No	3,504	84.8%
Self-esteem		
High	2,251	54.5%
Low	1,882	45.5%
HIV Knowledge		
High	3,066	74.2%
Low	1,067	25.8%
HIV stigma		
Fear		
High	3,298	79.8%
Low	835	20.2%
Moral		
High	2,939	71.1%
Low	1,194	28.9%
Personal		
High	2,983	72.2%
Low	1,150	27.8%
Community		
High	2,967	71.8%
Low	1,166	28.2%

Moreover, we found that 54.5% of the participants had high self-esteem, as presented in Table 1.

### HIV knowledge

Regarding knowledge on HIV, 74.2% of the participants were found to have a robust understanding (Table 1). In the eight items on HIV knowledge, the highest awareness rate was for the item “After the occurrence of high-risk behavior, one should take the initiative to seek AIDS counseling and testing” (92.4%) and the lowest was for “The main way of HIV transmission is male homosexual sex among China's students” (62.8%; Table 2).

**Table 2:** Distribution of HIV/AIDS related knowledge (*N* = 4,133).

Characteristic	Correct response (%)		
	Overall	Female (*n* = 2,549)	Male (*n* = 1,584)
Correct use of condoms may reduce the risk of spreading HIV.	84.6	81.4	89.7
HIV-infected person cannot be recognized from his/her appearance.	74.3	73.0	75.9
HIV is a serious incurable infectious disease.	69.3	68.5	70.5
The main mode of HIV transmission is male homosexual sex in China's students.	62.8	64.7	59.7
The use of new drugs (e.g., Meth, Ecstasy, K-powder, etc.) may increase the risk of HIV infection.	79.0	78.8	79.2
After the occurrence of high-risk behavior should take the initiative to seek AIDS counseling and testing.	92.4	92.9	91.7
The rights and interests of HIV infected persons, such as marriage, employment and school enrollment, are protected by Chinese laws.	71.9	70.7	73.9
Daily life and study contact will not be infected with HIV.	87.0	87.3	86.5

### HIV stigma

As indicated in Table 1, stigma, in terms of fear of casual transmission (79.8%), moral judgment (71.1%), personal stigma (72.2%), and perceived community stigma (71.8%) was largely prevalent.

### Factors associated with HIV knowledge

Factors including sociodemographic characteristics, individual behavior, HIV testing experience, access to HIV epidemic information, and self-esteem. Through univariate analysis, seven variables (gender, ethnicity, grade, only-child or not, sexual orientation, awareness of the HIV/AIDS epidemic in their region, and self-esteem) were found to be significantly related to HIV knowledge. After adjusting for these variables, six factors were significantly associated with HIV knowledge levels, and the associations were in the expected directions (Table 3).

**Table 3:** Multiple variate logistic regression analysis of factors associated with HIV knowledge.

Characteristic	AOR (95% CI)
Gender	
Male	1
Female	0.85 (0.73–0.98)^*^
Ethnicity	
Han	1
Others	0.71 (0.57–0.89)^**^
Grade	
Freshmen	1
Non-freshmen	1.37 (1.19–1.58)^***^
Sexual orientation	
Heterosexuals	1
Non-heterosexuals	0.77 (0.62–0.94)^*^
Whether aware of the HIV/AIDS epidemic in their region	
No	1
Yes	1.76 (1.42–2.20)^***^
Self-esteem	
Low	1
High	1.41 (1.22–1.62)^***^

^*^P < 0.05, ^**^P < 0.01, ^***^P < 0.001.

### Factors associated with HIV stigma

To determine statistically significant variables that influenced the four stigma dimensions, we performed a separate logistic regression analysis. As shown in Table 4, higher stigma levels for all four dimensions were always linked to insufficient knowledge regarding HIV. The discriminatory attitude among females was less than that in males in the three dimensions of moral judgment (AOR = 0.58; 95% CI = 0.50–0.67), personal stigma (AOR = 0.77; 95% CI = 0.67–0.89), and perceived community stigma (AOR = 0.65; 95% CI = 0.56–0.75). Similarly, participants with high self-esteem had less discriminatory attitudes in three dimensions: fear of casual transmission, personal stigma, and perceived community stigma (AOR = 0.81, 95% CI = 0.69–0.94; AOR = 0.79, 95% CI = 0.69–0.92; AOR = 0.62, 95% CI = 0.54–0.72, respectively). However, more students of other ethnicities expressed their fear of infection through casual contact (AOR = 1.38; 95% CI = 1.05–1.62), had negative judgment (AOR = 1.29; 95% CI = 1.02–1.64), and indicated personal stigma (AOR = 1.37; 95% CI = 1.07–1.76). All student except freshmen were more afraid of casual transmission (AOR = 1.17, 95% CI = 1.01–1.37), had experienced more personal stigma (AOR = 1.52; 95% CI = 1.32–1.75), and had experienced more community stigma (AOR = 1.85; 95% CI = 1.61–2.13) than freshmen. The participants who were aware of the HIV/AIDS epidemic in their region showed less fear of infection through casual contact (AOR = 0.74; 95% CI = 0.61–0.91) and lower levels of perceived community stigma (AOR = 0.83; 95% CI = 0.69–0.99). Non-heterosexuals were less likely to have negative moral judgment (AOR = 0.70; 95% CI = 0.57–0.86) (Table 4).

**Table 4:** Multiple variate Logistic regression analysis of factors associated with different dimensions of HIV stigma.

Characteristic	AOR (95% CI)			
	Fear	Moral	Personal	Community
Gender				
Male		1	1	1
Female		0.58 (0.50–0.67)^***^	0.77 (0.67–0.89)^**^	0.65 (0.56–0.75)^***^
Ethnicity				
Han	1	1	1	
Others	1.38 (1.05–1.82)^*^	1.29 (1.02–1.64)^*^	1.37 (1.07–1.76)^*^	
Grade				
Freshmen	1		1	1
Non-freshmen	1.17 (1.01–1.37)^*^		1.52 (1.32–1.75)^***^	1.85 (1.61–2.13)^***^
Only-child or not				
No			1	
Yes			0.82 (0.70–0.95)^**^	
Sexual orientation				
Heterosexuals		1		
Non-heterosexuals		0.70 (0.57–0.86)^**^		
Whether aware of the HIV/AIDS epidemic in their region				
No	1			1
Yes	0.74 (0.61–0.91)^**^			0.83 (0.69–0.99)^*^
Self-esteem				
Low	1		1	1
High	0.81 (0.69–0.94)^**^		0.79 (0.69–0.92)^**^	0.62 (0.54–0.72)^***^
HIV Knowledge				
Low	1	1	1	1
High	0.50 (0.41–0.61)^***^	0.61 (0.52–0.72)^***^	0.37 (0.31–0.45)^***^	0.43 (0.36–0.51)^***^

^*^P < 0.05, ^**^P < 0.01, ^***^P < 0.001.

## Discussion

This study primarily aimed to assess the level of knowledge regarding HIV and factors related to HIV stigma among college students to use in the development of HIV prevention strategies and better acquaint them with the additional benefits of approaches to HIV risk reduction, provided fundamental data critical for strengthening emergency management of infectious diseases in higher education institutions (HEIs). Our findings showed that college students had low HIV comprehension and high stigmatizing attitudes, which are consistent with that of studies conducted in other countries or regions (1, 9, 21, 24).

### HIV knowledge

The HIV knowledge level of respondents was 74.2%, which was similar to other studies in China (25–27). The results showed that there was no significant difference between male and female students overall regarding the level of HIV knowledge. However, it is worth noting that female students were significantly less aware of proper condom use to reduce the risk of HIV transmission. In addition, a higher proportion of male students were unaware that the main way of HIV transmission is male homosexual sex in students in China compared to their female counterparts. This poor basic knowledge about HIV is a likely the reason why youth people engage in risky sexual behaviors and why HIV infection rates are high among them (28).

Our study showed an adjusted association between knowledge of HIV and gender, ethnicity, grade, sexual orientation, awareness of the HIV/AIDS epidemic, and self-esteem. These associations were similar to results found with other college students (26, 27). Therefore, more targeted promotion activities and interventions are needed in colleges to increase the level of HIV knowledge. It is also important to determine the level of HIV-related knowledge, identify disparity, and implement situational education and skill-enhancing HIV prevention methods, although knowledge on HIV may not ultimately lead to changes in risky behavior and utilization of prevention strategies (9).

### HIV stigma

In terms of HIV stigma, the overall stigma levels were generally high in this study. The findings indicated that high levels of knowledge across all four dimensions were associated with less stigma. Seven other variables, namely, gender, ethnicity, grade, only-child or not, sexual orientation, awareness of the HIV/AIDS epidemic, and self-esteem, played a different role in influencing the various aspects of HIV stigma.

This study confirms the fact that individuals with higher understanding of HIV tend to have lower stigma (9, 29). Sexual orientation was also significantly correlated with HIV stigma. Non-heterosexual students had less moral judgment regarding HIV stigma. This situation may account for diverse sexual identities and intersectional connections with HIV infection in the dimension of social identification. Such results demonstrated the need to enhance HIV-associated knowledge and reduce stigma through targeted interventions.

In agreement with previous studies, females were less likely to express the idea of stigma with HIV (21, 30). Females showed increased compassion, which was mainly due to gender roles, as women are economically, culturally, and socially disadvantaged and have more housework and childcare responsibilities than men, especially in China (21).

Our data suggest that participants who were aware of the HIV/AIDS epidemic and had a high self-esteem were unlikely to have stigmatizing attitudes. This conforms to the knowledge–attitude–belief–practice model, proving that high self-esteem and increased knowledge about HIV can reduce misunderstandings in terms of HIV transmission of through casual contact and ultimately help reduce HIV-related stigma (21).

Surprisingly, being an only child significantly reduced personal stigma. An explanation of this phenomenon could be that an only-child usually has higher self-rating than children with siblings (31), which may result in an accurate perception of personal risk.

Additionally, our research found that in addition to moral judgment, non-freshmen always showed a higher degree of stigma across other three dimensions. This may be partly because higher grade (age) undergraduates were more possibly to engage in unsafe sex despite their higher level of knowledge, such as by less use of condoms. University students are less controlled by parents over time and also have more opportunities of interacting with peers of the same or opposite gender (21, 32). As a result, non-freshmen students showed more stigma against HIV-infected people to reduce discomfort associated with unsafe sex (32).

Overall, stigma is a complex issue, which cannot be rooted in simple causality (1). Therefore, there is a need for multilayered intervention at the individual, interpersonal, community, institutional, and structural levels to reduce stigma of HIV among college students.

## Conclusion

Our results showed that college students in southwest China have a low level of HIV knowledge and a high degree of HIV stigma. That directly compromise institutional capacities for effective infectious disease emergency management. The results also emphasize the importance of focusing on HIV knowledge promotion among college students to combat HIV-related stigma. Further studies are required to find effective ways to increase the level of HIV knowledge to decrease stigma among college students. As personal self-esteem heavily influenced HIV knowledge and stigma, students need to be trained with targeted learning and practice opportunities to improve their self-esteem. These dimensions should be addressed independently when developing new interventions because the four aspects of HIV stigma (fear, moral judgment, personal stigma, and perceived community stigma) were influenced by different factors.

## Data Availability

The raw data supporting the conclusions of this article will be made available by the authors, without undue reservation.
